# 

**Published:** 1881-12

**Authors:** 


### INDEX TO VOLUME XLIII.

PAGE.

A.

Abadie, Ch., M.D., Glaucoma... 549
Advancement of the Rectus, A. E. Prince, M.D.............. 229
Albumen in Urine, Ed. Robin.. 652
Alcohol, Uses and Abuses of, Report by E. Ingals, M.D., Illinois State Medical Society... 80
Alumni Prize................. 96
Amaurosis, Quinine, H. Knapp, M.D .......................... 552
American Dermatological Association, Fifth Annual Meeting —Official Report of, A. Van Harlingen, M.D. ............. 507
Anatomy, Minute, of the Skin, Charles Heitzmann, M.D.......523
Andrews, Prof. E., Surgical Clinic....................... 71
Andrews, E., M.D., Course of the Bullet in the President's Wound.................... 180
Andrews, E., M.D., Surgical Clinic........................ 398
Aneurism, Diffuse Idiopathic, by G. Frank Lydston, M.D.... 132
Animal Heat, Paper on, Dr. J. H. Hollister, Illinois State Medical Society............ 79
Ainhum, Examination of, Dr. Heitzmann ................. 631
Anthropological Studies....... 558
Anthrax, Vaccinating for.. ... 435
Aspiration in Distended Gall Bladder ................... 420
Auricle Bilateral, Rudimentary, H. Knapp, M.D.............. 553
Auricles, Operation for Prominence of, Ed. T. Ely, M.D..... 553

B.

Ball, Prof., Syphilitic Insanity. 437
Bandage, Roller, Nelson H. Church, M.D................ 396
Bartholow, Roberts, M.D., Treatment of Consumption....... 665
Bayard & Wilson, M.D., Special Report on Leprosy.......... 622
Beaconsfield's Doctors......... 605

PAGE.

Belfield, W. T., London Letter....... .............. 416, 635
Birge, Prof. E. A., Notes on Crustacea in Chicago Water. 584
Bladder, Irritable in Women, J. H. Etheridge, M.D........... 463
Blindness, Color, During Pregnancy, E. L Holmes, M.D.... 606
Books and Pamphlets Received. 95
Botany, Medical, of Southern Illinois, J. M. G. Carter, M.D......................358, 590
Bridge, Norman, M.D., Case of Hydrophobia .............. 402
Brown, Geo. Preston, Sewer Gas and its Dangers............. 221
Buccal Ulcerations of Constitutional Origin, Edward Wigglesworth, M.D ... ......... 616
Bulkley, L. Duncan, M.D., The Skin in Health and Disease.. 93

C.

Cadaveric Alkaloids .......... 445
Calcium, Oxy-sulphuret of, Clinical Experience in, C. Heitzmann, M.D.............. 626
Cancer of the Larynx, E. F. Ingals, M.D.................... 9
Carapace, Formation of, Prof. E. A. Birge................. 584
Cariatides from Cadavers...... 555
Carter, J. M. G., M.D., Medical Botany of Southern Illinois.. 358, 591
Catheter, A novel way to use a, Edward C. Huse, M.D........ 201
Causes of Consumption, Study of, Dr. Edward Playter...... 73
Cautery, The Actual, T. F. Frank, M.D................. 343
Cerebral Meningitis, by James Hutchinson.............. ... 225
Cerebro Spinal Meningitis, Post Mortem Evidences.......... 228
Charney, W., M.D., New Formation of Blood Vessels in the Vitreous .................. 548

PAGE.
Chian Turpentine, Paper on, by E. Andrews, M.D., Illinois State Medical Society........ 81
Chicago Medical Society, Dr. E. Ingals, President—Society Meetings..................86, 205
Chorea, Paralytic, Dr. Gowers.. 554
Church, Nelson H., M.D., A Bandage Roller............. 396
Clevenger, S. V., M.D., Medical Electricity ................. 449
Clinical Reports.............. 71
Clinical Contributions, by E. L. Holmes, M.D................ 551
Color Blindness during Pregnancy, E. L. Holmes, M.D.... 606
Committee on Dermatology, Illinois State Medical Society... 78
Consumption, Study of Causes of, Dr. Edward Playter...... 73
Consumption, Treatment of, Roberts Bartholow, M.D...... 665
Correspondence, Domestic..... 421
Correspondence, Foreign, E. S. McKee, M.D................ 405
Craniotomy—Is it Ever Justifiable, A. Reeves Jackson, M.D.. 337
Cremation, Article on, C. W. Purdy, M.D..............188, 264
Crothers, T. D., M.D., Etiology of Inebriety.................. 471
Crustacea in Chicago Water, Prof. E. A. Birge............ 584
Crystaline Lenses, Dislocation of by kick of a Horse, J. C. Wordsworth, F.R.C.S......... 547
Cutaneous and Venereal Memoranda, H. G. Piffard, M.D.... 94
Cyanosis, A Case of, M. H. Sears, M.D................. 479
Cystic Tumors of Larynx, E. F. Ingals, M.D................. 23

D.

Death of President Garfield.... 426
Death Rate, Annual per 1,000 in chief cities of the U. S....... 88
Dementia, Syphilitic.......... 439
Dermatology, Committee on, Report of, Illinois State Medical Society..................... 78
Dermatological Association, American, Official Report, A. Van Harlingen ............. 507
Dermatology and its Relations to Periodical Literature, James Nevins Hyde, M.D.... ..... 507
Diseases of the Eye and Ear, W. F. Mittendorf, Review of. ... 92

PAGE.
Diseases of the Nervous System, S. Wier Mitchell, M.D., Review ........................ 325
Diseases of the Skin, Duhring, Review .................... 320
Doctors, Beaconsfield's........ 605
Doering, E. J., M.D., New Medical Colleges................ 639
Domestic Correspondence, E. J. Doering, M.D................ 639
Duhring, Louis A., M.D., A Small Pustular Scrofuloderm.... .. 529

E.

Earle, Chas. W., M.D., Inebriety, Etiology and Treatment..... 236
Eclampsia, Puerperal Convulsions, by H. Z. Gill, M.D..... 113
Eclampsia, D. A. Walden, M.D.. 356
Editorials..................97, 424
Editorials, Late, in the Medical Press...................... 103
Electricity, Medical, S. V. Clevenger, M.D................. 449
Electricity, Medical, W. J. Morton, M.D..................... 643
Ely, Ed. T., M.D., Operation for Prominence of the Auricles.. 553
Enucleation of Eye in Case of Epilepsy, H. Rosencrans, M.D. 542
Epilation, Paper on, by P. S. Hayes, M.D., Illinois State Medical Society............. 81
Epilepsy, Cure of Case by Enucleation of Eye, H. Rosencrans, M.D.................. 542
Epithelioma of Lower Eyelid, George Lawson, F.R.C.S...... 548
Etheridge, J. H., M.D., Treatment of Irritable Bladder in Women ..................... 463
Etiology of Inebriety, T. D. Crothers, M.D .................. 471
Extra Uterine Fœtation, H. Rosencrans, M.D................ 178
Eyelash in Anterior Chamber, E. L. Holmes, M.D........... 607

F.

Face, Fistula upon the, E. S. Talbot, M.D................. 604
Fibro Cellular Tumors, E. F. Ingals, M.D ... ............. 22
Fibroid Tumor of Larynx, E. F. Ingals, M.D................ 21
Fibromata of Larynx, E. F. Ingals, M.D..................... 21
Fistula Upon the Face, E. S. Talbot, M.D................. 604
Fœtation, Extra Uterine....... 421

PAGE.

Foreign Correspondence, W. T. Belfield, M.D. .......... 635
Fowler, R., M.D., Letter from Aurora .......... 220
Fox, L. Webster, M.D., Formation of Vessels in Vitreous... 548
Frank, G. F., M.D., The Actual Cautery .......... 343

G.

Gall-Bladder, Aspiration of.... 420
Gardiner, E. J., M.D., Quarterly Abstract of Ophthalmological and Otological Literature.... 546
Garfield, The Case of President, Frank H. Hamilton, M.D. .... 658
Gill, H. Z., M.D., Paper on Puerperal Convulsions.......... 113
Glaucoma, Ch. Abadie, M.D... 549
Gross, S. D., M.D., Nocturnal Incontinence of Children...... 401
Gunn, R. Marcus, M.D., Constant Electric Current in Atrophy of Optic Nerve.......... 546
Gynæcological Society, Chicago, Address of President, De Laskie Miller, M.D.......... 561

H.

Hamilton, M.D., Frank H., The Case of President Garfield... 658
Hayes, P. S., M.D., Paper on Epilation, Illinois State Medical Society.......... 81
Health, Illinois State Board of. 530
Health, Michigan State Board of. 609
Heitzmann, M.D., Charles, A Contribution to the Minute Anatomy of the Skin.....523, 575
Heitzmann, Charles, M.D., Use of Oxy-sulphuret of Calcium. 626
Heitzmann, C., M.D., Examination of a Specimen of Ainhum 631
Hermaphrodite, Dr. Magistos... 559
Holmes, E. L., M.D., Clinical Contributions .......... 551
Holmes, E. L., M.D., Color Blindness During Pregnancy...... 606
Holmes, E. L., M.D., Eye-Lash in Anterior Chamber.......... 607
Hotz, F. C., M.D., Malarial Keratitis .......... 598
Huse, Edward C., M.D., A Novel Way to Use a Catheter....... 201
Hutchinson, James, M.D., on Cerebral Meningitis......... 225
Hyde, James Nevins, M.D., Dermatology and its Relations to Periodical Literature........ 507

PAGE.

Hyde, J. Nevins, M.D., A Case of Acute Tubercular Leprosy... 613
Hydrophobia, Case of, Norman Bridge, M.D.......... 402

I.

Identity of Small-pox and Cowpox, by H. M. Lyman, M.D... 84
Illinois State Board of Health, Meeting of.......... 530
Illinois State Medical Society, Thirty-first Annual Meeting.. 74
Incontinence, Nocturnal, of Children, S. D. Gross, M.D ...... 401
Inebriety, Etiology and Treatment of, Chas. W. Earle, M.D. 236
Inebriety, Etiology of, T. D. Crothers, M.D.......... 471
Ingals, E. F., M.D., Laryngeal Tumors .......... 9
Ingals, E., M.D., Report on Uses and Abuses of Alcohol, Illinois State Medical Society... 80
Ingals, E., M.D., President Chicago Medical Society........ 86
Insanity, Syphilitic, Prof. Ball. 437
Items .......... 330

J.

Jackson, A. Reeves, M.D., Is Craniotomy Ever Justifiable? 337
Jurisprudence, Medical Progress in.......... 443

K.

Keratitis, Treatment of, Dr. Galezowski.......... 553
Keratitis, Malarial, F. C. Hotz, M.D .......... 598
Knapp, H., M.D., Quinine Amaurosis.......... 552
Knapp, H., M.D., Bilateral Rudimentary Auricle.......... 553
Knee Joint, Amputation of, E. Andrews, M.D.......... 398

L.

Labor, Management of, Dr. Rendu.......... 441
Lambert, J. W., M.D., Letter from Indiana.......... 89
Lawson, George, F.R.C.S., Epithelioma of Eyelid.......... 548
Lawson, George, M.D., Voluntary Nystagmus.......... 549
Leprosy, Acute Tubercular, J. N. Hyde, M.D.......... 613
Leprosy in Louisiana.......... 556

PAGE.
Leprosy, Tubercular, Case of, I. E. Atkinson, M.D ........... 612
Leprosy, Discussion on........ 614
Leprosy, Special Report on, Drs. Bayard and Wilson.... .... 622
Letter from Prescott, Wis...... 641
Listerism and Carbolic Acid, Paper on, C. Truesdell, M.D., Illinois State Medical Society 81
Listerism Tottering.......... 657
Litholapaxy, Prof. E. Andrews. 71
London Letter, W. T. Belfield, M.D........................ 416
Long, J., M.D., Report of Fistula Upon the Face............. 604
Lunatics, Criminal............ 444
Lyman, H. M., M.D., Vacination..................... 1
Lyman, H. M., M.D., President West Chicago Medical Society, Paper on Identity of Small-pox and Cow-pox........... 84
Lyman, H. M., M.D., Vacination..................... 483
Lydston, G. Frank, M.D., Diffuse Idiopathic Aneurism........ 132
Lymphangioma Tuberosum Cutis Multiplex, Case of, by A. Van Harlingen, M.D......... 528

M.

Malarial Keratitis, F. C. Hotz, M.D......................... 598
Massage, J. I. Tucker, M.D..... 394
Maternal Impressions, Case, West Chicago Medical Society, by H. D. Valin, M.D...... 87
Maternal Impressions—Letter, S. O. Stockschlager, M.D..... 313
McKee, E. S., M.D., The London Medical Man............... 415
Mease, L. A., M.D., Obituary Resolutions.... ............ 541
Medical Botany, Studies in, J. M. G. Carter, M.D.........358, 590
Medical Electricity, S. V. Clevenger, M.D.................. 449
Medical Electricity, W. J. Morton, M.D.................. 643
Medical Society, State of Illinois..................... 74
Medicine, Specialism in, Paper by A. Reeves Jackson, Illinois State Medical Society........ 79
Michigan State Board of Health, Report of....... ...203, 314, 609
Microscopical Society of Illinois, Meeting of............. 545

PAGE.
Miller, De Laskie, M.D., President, Address Before Chicago Gynæcological Society....... 561
Mittendorf, W. F., M.D., Diseases of the Eye and Ear, Review.. 92
Morton, W. J., M.D., Medical Electricity.................. 643

N.

Nervousness, the Result of Intemperance.... ............ 634
Nerve Resection and Stretching, Report Com. on Surgery, State Medical Society........... 76
Nervous System, Diseases of, S. Weir Mitchell...... ....... 325
Newkirk, A. B., Jr., M.D., Letter from Falls City............. 216
Normandy Letter............. 208
Numeration and Measurement, Paper by Dr. Weller........ 86
Nystagmus, Voluntary, George Lawson, F.R.C.S., N. Y........ 549

O.

O'Connell, P., M.D., Treatment of Pneumonitis....... ..... 29
O'Connell, P., M.D., New Umbilical Cord Repositor.......... 469
Ophthalmological and Otological Literature, Abstract of, E. J. Gardiner, M.D............. 546
Optic Nerve, Atrophy, Use of Galvanic Current in, R. Marcus Gunn, M.D.............. 546
Our Calling, Address by F. Wm. Schwan, M.D............... 260
Outgrowths from Vocal Cord, E. F. Ingals, M.D............... 13

P.

Pagenstecher, H., M.D., Extraction of Splinters of Iron from Vitreous Humor............ 550
Palmar Tendons, Anatomy of Sheaths of, Paper by Roswell Park, M.D., Ill. State Medical Society..................... 81
Pancreatic Microzymes, Poisonous Effect of................ 207
Papillary Growths of Larynx, E. F. Ingals, M.D............. 15
Park, Roswell, Paper on Anatomy of the Sheaths of the Palmar Tendons, Illinois State Medical Society............. 81
Park, Roswell, M.D., Report of One Hundred Cases of Rötheln 130
Pathology and Treatment of Yellow Fever, H. D. Schmidt, M.D. 36, 135, 273, 364, 487

PAGE.

Picard, Geo. H., M.D., Pathology of Involuntary Spermatic Fluxes ..................... 252
Piffard, H. G., M.D., Cutaneous and Venereal Memoranda.... 94
Pneumonitis, Treatment of, P. O'Connell, M.D.............. 29
Practice of Medicine, Legal Regulation of, Paper by M. A. McClelland, M.D., Illinois State Medical Society........ 80
President's Wound, The Course of the Bullet in, E. Andrews, M.D .......................... 180
Prince, A. E., M.D., Advancement of the Rectus................ 229
Prize, Alumni................. 96
Prize, Hammond, The........ 110
Prize, Warren Triennial, The.. 102
Prize Essay............... .... 397
Prize Questions............... 557
Puerperal Convulsions, D. A. Walden, M.D... ............. 356
Purdy, C. W., M.D., Cremation, Article on...............188, 264

Q.

Quinine Amaurosis, H. Knapp, M.D .......................... 552

R.

Rachitis and Calcium Phosphate, Dr. DesVallères....... 222
Regulation of Practice of Medicine, Paper by M. A. McClelland, M.D., Ill. State Medical Society ................. ... 80
Repositor, New Umbilical Cord, P. O'Connell, M.D........... 469
Report, Official, of American Dermotological Association, A. VanHarlingen, M.D....... 507
Reports, Clinical, Prof. Andrews 398
Reports, Hospital, H. D. Valin, M.D. ...................... 402
Resection of Small Intestine.... 634
Reviews and Book Notices:
Manual of Diseases of the Eye and Ear, by W. F. Mittendorf, M.D................. 92
The Skin in Health and Disease by Duncan Bulkley, M.D .................... 93
Cutaneous and Venereal Memoranda, by Henry Piffard, M.D ..................... 94
Sewer Gas and its Dangers, by George Preston Brown.... 221
Diseases of the Skin, by Louis A. Duhring, M.D........... 320

PAGE

Lectures on Diseases of the Nervous System, by S. Weir Mitchell, M.D............. 325
Antagonism Between Medicines, Roberts Bartholow, M.D ..................... 429
On the Bile, Jaundice and Bilious Diseases, by J. Wickham Legg, F.R.C.P.... 430
Medical Electricity, by Roberts Bartholow............ 432
Hernia, Strangulated and Reducible, Joseph H. Warren, M.D ..................... 433
Lectures on Diagnosis and Treatment of Diseases of Chest, Throat, and Nasal Cavities, E. Fletcher Ingals, M.D ..................... 543
The Diseases of Children, Wm. Henry Day.......... 650
Cyclopædia of Medicine, Ziemssen, vol. ix.......... 651
Rima Glottidis, Method of Removing Foreign Bodies from. 423
Robin, Ed., Albumen in Urine. 652
Rosencrans, H., M.D., Extra Uterine Fœtation............. 178
Rosencrans, H., M.D., Cure of Case of Epilepsy........... 542
Rötheln, One Hundred Cases of, Roswell Park, M.D.... ...... 130

S.

Sanitation Railroad...... .... 583
Schmidt, H. D., M.D., Yellow Fever, Pathology and Treatment.......36, 135, 273, 364, 487
School, Training, for Nurses, Washington................ 111
Schwan, F. Wm., M.D., Our Calling, Address before Seneca Co. Medical Society......... 260
Scrofuloderm, A Small Pustular, Louis A. Duhring, M.D...... 529
Sears, M. H., M.D., a Case of Cyanosis .................. 479
Sewer Gas and Its Dangers, Geo. Preston Brown......... 221
Skin, The, In Health and Disease, L. Duncan Bulkely, M.D. 93
Skin, A Contribution to the Minute Anatomy of, Charles Heitzmann, M.D............ 523
Skin, Minute Anatomy of, C. Heitzmann, M.D............ 575
Society, Medical, of Illinois.... 74
Society, Chicago, Gynæcological, Address of President, DeLaskie Miller, M.D......... 56

PAGE.
Society, West Chicago Medical. 84
Specialism in Medicine, Paper by A. Reeves Jackson, Illinois State Medical Society....... 79
Spermatic Fluxes, Involuntary, Pathology of, Geo. H. Picard, M.D ...................... 252
Stockschlager, S. O., M.D, Letter, Maternal Impressions....... 313
Summer Resorts, Dangers of... 538
Surgical Clinic of Prof. E. Andrews ...................... 71
Syphilitic Insanity, Prof. Ball.. 437

T.

Talbott, Eugene S., M.D., Fistula Upon the Face............. 604
Taylor, John J., Letter from Streator ...................... 219
Therapy, Internal, Limitations of, in Skin Diseases, J. C. White, M.D................. 525
Treatment of Pneumonitis, P. O'Connell, M.D. ............ 29
Treatment of Wounds, Report Committee of Surgery, State Medical Society............ 75
Treatment of the Irritable Bladder in Women, J. H. Etheridge, M.D................... 463
Treatment and Pathology of yellow Fever, H. D. Schmidt, M.D. 36, 135, 273, 364, 487
Treatment of Consumption, Roberts Bartholow, M.D......... 665
Treves, F.R.C.S., Frederick, Tubercle, its Histological Characters ...................... 670
Truesdell, C., M.D., Paper on Listerism and Carbolic Acid, Ills. State Medical Society.... 81
Tubercle, its Histological Characters, Frederick Treves, F.R.C.S. ...................... 670
Tubercular Leprosy, A Case of, I. E. Atkinson, M.D.......... 612
Tuberculosis, Infectiousness of, Dr. H. Gradle, Paper on, Ills. State Medical Society....... 80
Tucker, M.D., J. I., Massage.... 394
Tumors, Laryngeal, E. F. Ingals, A.M., M.D.................... 9
Tumors, Fibrocystic of Larynx, E. F. Ingals, M.D........... 9
Tumors, Fibro Cellular, E. F. Ingals, M.D..... ........ .... 22
Turpentine, to Prevent Vomiting Under Ether, E. Andrews, Clinic of. .................. 73

PAGE.

U.

Umbilical Cord Repositor, P. O'Connell, M.D.............. 469
Urine, Albumen in, Ed. Robin.. 652
Uterine Fœtation, Extra........ 421

V.

Vaccinating for Anthrax, by Pasteur .................... 435
Vaccination, Henry M. Lyman, M.D .......... ......... ... 483
Vaccination, Thos. F. Wood, M.D .......... ............ 347
Valin, H. D., M.D., Hospital Reports ...................... 402
Valin H. D., M.D., West Chicago Medical Society, Maternal Impressions Case............ 87
Van Harlingen, A., M.D., Official Report of Fifth Annual Meeting American Dermatological Association .... ........... 507
Van Harlingen, A., M.D., A Case of Lymphangioma Tuberosum cutes multiplex........ 528
Varicocele, Report Committee on Surgery, State Medical society ..................... 76
Vienna Letter.................... 213
Vitreous Humor, Extraction of Splinters of Iron from, H. Pagenstecher, M.D........... 550
Vocal Cords, Outgrowths from, E. F. Ingals, M.D............ 13

W.

Walden, D. A., M.D., Eclampsia. 356
Weller, Dr., Paper on Numeration and Measurement......... 86
West Chicago Medical Society. 84
White, J. C., M.D., the Limitations of Internal Therapy in Skin Diseases................ 525
Wigglesworth, E., M.D., Buccal Ulcerations ................ 616
Wilson & Bayard, Special Report on Leprosy..... . .... 622
Wood, Thos. F., M.D., Vaccination ...................... 347
Wordsworth, J. C., M.D., Case of Dislocation of Both Crystaline lenses ...................... 547
Wounds, Treatment of, Committee on Surgery, State Medical Society .................... 75

Y.

Yellow Fever, Pathology and Treatment of, H. D. Schmidt, M.D.........36, 135, 273, 364, 487

SOCIETY MEETINGS.

Chicago Medical Society—Mondays, Dec. 5-19.
West Chicago Medical Society—Mondays, Dec. 12-26.
Biological Society—Wednesday, Dec. 7.

CLINICS.

MONDAY.

Eye and Ear Infirmary—2 p. m., Ophthalmological, by Prof. Holmes; 3 p. m., Otological, by Prof. Jones.

Mercy Hospital—2 p. m., Surgical, by Prof. Andrews.

Woman's Medical College—2 p. m., Dermatological and Venereal, by Prof. Maynard; 3 p. m., Diseases of the Chest, Prof. Ingals.

TUESDAY.

Rush Medical College—3 p. m., Dermatological and Venereal, by Prof. Hyde.

Cook County Hospital—2 to 4 p. m., Medical and Surgical Clinics.

Mercy Hospital—2 p. m., Medical, by Prof. Quine.

WEDNESDAY.

Chicago Medical College—2 p. m., Eye and Ear, by Prof. Jones.

Rush Medical College—2 p. m., Medical, by Dr. Bridge; 3 p. m., Ophthalmological and Otological, by Prof. Holmes; 3:30 to 4:30 p. m., Diseases of the Chest, by Dr. E. Fletcher Ingals.

THURSDAY.

Chicago Medical College—2 p. m., Gynæcological, by Prof. Jenks.

Rush Medical College—2 p. m., Diseases of Children, by Dr. Knox; 3 p. m., Diseases of the Nervous System, by Prof. Lyman.

Eye and Ear Infirmary—2 p. m., Ophthalmological, by Dr. Hotz.

Woman's Medical College—3 p. m., Surgical, by Prof. Owens.

FRIDAY.

Cook County Hospital—2 to 4 p. m., Medical and Surgical Clinics.

Mercy Hospital—2 p. m., Medical, by Prof. Davis.

SATURDAY.

Rush Medical College—2 p. m., Surgical, by Prof. Gunn; 3 p. m., Orthopœdic, by Prof. Owens.

Chicago Medical College—2 p. m., Surgical, by Prof. Isham; 3 p. m., Neurological, by Prof. Jewell.

Woman's Medical College—11 a. m., Ophthalmological, by Prof. Montgomery; 2 p. m., Gynæcological, by Prof. Fitch.

Daily Clinics, from 2 to 4 p. m., at the Central Free Dispensary, and at the South Side Dispensary.

